# Inhibition and individual differences in behavior and emotional regulation in adolescence

**DOI:** 10.1007/s00426-021-01565-8

**Published:** 2021-08-07

**Authors:** Chiara Malagoli, Carlo Chiorri, Laura Traverso, Maria Carmen Usai

**Affiliations:** Department of Education, University of Genoa, C.so Andrea Podestà 2, 16128 Genova, Italy

## Abstract

The Impulsivity/Reflexivity issue in inhibitory control ability has seldom been investigated in terms of individual differences in typically developing populations. Although there is evidence of changes in executive functioning (EF), including inhibition, in adolescence, very little is known about the role of individual differences. Using the data from 240 14-to-19-year-old high school students who completed a battery of EF tasks (Flanker, Go No-Go, Antisaccade, and Stop signal task), measures of emotion regulation strategies and behavioral difficulties, we performed a latent profile analysis to identify qualitatively distinct score profiles. The results showed the existence in adolescence of two inhibition profiles, Impulsive *vs* Reflexive, differing in performances at the inhibition tasks. The two profiles were not associated with socio-demographic characteristics, or to psychological variables, such as behavioral characteristics and emotional regulation strategies.

**Supplementary Information:**

The online version contains supplementary material available at 10.1007/s00426-021-01565-8.

Inhibition is one of the core abilities of Executive Function (EF), a set of top-down processes that allow people to regulate their thoughts and behaviors (Miyake & Friedman, 2012). Inhibition includes different behavioral and cognitive abilities, such as managing impulses/interferences (Diamond, 2013; Nigg, 2000), and fosters self-regulation also in complex situations.

EFs have a prolonged development over time, from early infancy until late adolescence up to young adulthood (Best & Miller, 2010; Johnson, 2000, 2001; Luna et al., 2004), and inhibition emerges early, showing a significant peak of development in preschool age (see e.g., Best & Miller, 2010). Changes in their latent organization also occur early on in development and separated inhibitory dimensions, such as the ability to stop a prepotent response and the ability to suppress the stimuli interference, can be already observed at age 3 (Gandolfi et al., 2014). This structure appears to be stable during childhood (Traverso et al., 2018) and adulthood (Rey-Mermet et al., 2017). Changes in the EF domain are well documented also in adolescence (Cragg & Nation, 2008; Crone et al., 2006; Huizinga et al., 2006; Michel & Anderson, 2009), but very little is known about the existence of different profiles in terms of inhibitory efficiency and how they may be related to other cognitive and emotional domains between age 14 and 19. Since during adolescence a tendency toward impulsiveness and risk-taking/sensation seeking (Dahl, 2004) co-occur with biologically based changes of ER, and a lack of control has been associated with maladaptive and dangerous behaviors (substance use and gambling, Vitaro et al., 1998; internet addiction, Cao et al., 2007; earlier onset of alcohol-use, Soloff et al., 2010), evidence of different profiles in inhibition in adolescence can be of paramount importance for understanding development. More specifically, it seems necessary to investigate whether it is possible to distinguish diverse inhibitory profiles in adolescence and to test whether these differences in inhibitory skills are related to emotional and behavioral difficulties.

## Inhibition and individual differences

The lack of control in inhibition has recently been theorized as a distinctive pattern of behavior (DeYoung, 2010). Nevertheless, very little is known about the development of specific individual profiles of inhibition in typical development.

To the best of our knowledge, very few studies have investigated the existence of different profiles in cognitive inhibition. Some research about behavioral inhibition, see the “Dual Process” theorization in decision making (e.g., Evans & St, 2003), has been carried out, and focused on the neurobiological bases of environmental and social interactions, together with resource management in complex situations. However, little is known about the possibility to profile core cognitive abilities that are specific components of the general construct of inhibition, such as response inhibition (e.g., the ability withhold a response), interference control (e.g., the ability to manage distracting or interferent stimuli), oculomotor inhibition (e.g., execute a subdominant response in the face of a more dominant response), and, in general, more specific abilities connected to cognitive and behavioral inhibition (Nigg, 2000). Very few studies have tested the existence of different profiles in terms of Impulsivity *vs* Reflexivity regarding executive functioning (Haghighi et al., 2015; Quiroga et al., 2007). In particular, Haghighi et al. (2015) investigated tendencies toward Impulsivity/Reflectivity in typical development using the Matching Familiar Figures Test in college students. By analyzing both the balance and the imbalance between accuracy and RTs, the authors found two distinct profiles, supporting the existence of different response control tendencies, impulsive *vs* reflective.

Schiller et al. (2014) investigated the development of individual differences in inhibitory capacity through an electrophysiological index of response inhibition, the No Go-Anteriorization (NGA; Fallgatter & Strik, 1999). Their results indicated that higher bilateral baseline activation in the lateral prefrontal cortex (PFC) was associated with a larger NGA, which is a better inhibition response. Although the authors suggested caution in interpreting these results, since the EEG slow waves at rest are complex to interpret, this finding supports the role of PFC in response inhibition. The hypothesis about differences in the baseline of these processes is consistent with the existing behavioral literature (Haghighi et al., 2015; Quiroga et al., 2007).

The recent studies on EFs have suggested that distinct patterns of neuropsychological functioning may exist in both hyperactive/inattentive and healthy adolescents and young adults (Gomez et al., 2014; Rau et al., 2016). Specifically, these studies hypothesized that homogenous subgroups of participants could be identified on the basis of patterns in the performance of a set of EF tests (e.g., Gomez et al., 2014). Rau et al. (2016) observed three distinct profiles characterized by (i) average EF performances, (ii) set maintenance weakness under nonverbal conditions, and (iii) weaknesses in cognitive flexibility combined with poor executive performance. Quite recently, it has been debated as well whether neuropsychological tests are necessarily a better means for assessing impulsivity than are trait measures, suggesting that cognitive impulsivity might only sometimes be indicative of trait impulsivity, and trait impulsivity might also only sometimes be indicative of cognitive impulsivity, but without a complete overlap (Glicksohn et al., 2016). Glicksohn et al. (2016) proposed to incorporate both self-report and analysis of performance in neuropsychological assessment research, as a solution to such incomplete overlap and the related difficulties in assessing impulsivity. To the best of our knowledge, no similar study has been performed using a variety of inhibitory tasks to investigate different profiles in inhibitory baselines using a person-centred approach.

## Inhibition and emotion regulation

Emotion regulation (ER) can be conceptualized as the ability of controlling and regulating one’s emotions (Eisenberg et al., 2010; Mischel & Ayduk, 2002). According to Diamond (2013), ER is a component of the broader concept of self-regulation and substantially overlaps with inhibitory abilities. Inhibition supports self-regulation even in more emotional conditions and it plays a central role in regulating behavior (Eisenberg et al., 2010; Prencipe et al., 2011). Specifically, response inhibition influences adaptive abilities and self-regulation (Prencipe et al., 2011), and, consistent with these findings, impulsivity has been associated with maladaptive/dangerous behaviors (Cao et al., 2007; McLaughlin et al., 2011; Soloff et al., 2010; Vitaro et al., 1998). In this perspective, being efficient or impulsive as a “baseline” may play a role in differences in the ability to be adaptive despite the emotional arousal.

The role of inhibition in ER was also highlighted by Schweizer et al. (2020), who reviewed the literature on adolescents performing the same cognitive control tasks (Go-No Go or Stroop task) with affective vs. neutral stimuli. Inhibition of affective stimuli was found to display a similar but more protracted time-course linear developmental trajectory with respect to inhibition of neutral stimuli (Tottenham Hare, & Casey, 2011) or a quadratic effect of development (e.g., Cohen et al., 2016; Somerville et al., 2011), suggesting that inhibition of affective information is reduced during adolescence. The authors also assumed that inhibition of affective information is associated with ER, showing that the use of maladaptive strategies such as rumination can be associated with cognitive control. In particular, adolescents with a strong tendency to rely on rumination showed poorer performance in inhibition to affective stimuli (Hilt et al., 2014, 2017; Romens & Pollak, 2012). The literature suggests a link between the lack of inhibitory abilities and the presence of difficulties in ER; however it is unclear whether specific individual differences in inhibitory abilities may be associated with a difference in the ability to regulate emotions.

## Present study

The first aim of the current study was to identify different profiles of inhibitory efficiency, with a cross-sectional design, in a sample of adolescents using a battery of computerized tasks designed to assess different components of inhibition, such as response inhibition (i.e., the ability to suppress a dominant but inappropriate response and to prevent impulsive behavior), interference control (i.e., the ability to prevent interference due to resource or stimulus competition), and oculomotor inhibition (i.e., the ability to inhibit saccades towards to-be-ignored stimuli, and in this perspective to execute a subdominant response in the face of a more dominant response; Diamond, 2013; Friedman & Miyake, 2004; Nigg, 2000). The second aim of the study was to investigate whether such profiles of inhibition efficiency were associated with individual differences in ER and behavioral outcomes.

Previous research (Rothbart et al., 2003; Zelazo & Müller, 2002) has shown a protracted development of inhibition up to late adolescence. Owing to the high sensitivity of EF to the environment (Mezzacappa, 2004; Noble et al., 2005), we expected inhibition to be sensitive to individual differences development, in terms of efficiency, intended in the balance of both speed, measured throughout RTs, and accuracy, and that these differences would emerge even in the typical population (Diamond, 2002; Kochanska et al., 1997). In line with the few studies in this domain (Haghighi et al., 2015; Quiroga et al., 2007), we expect to be able to identify at least two different profiles, among the possible combinations, inefficient, efficient, or reflexive based on the balance or unbalance between accuracy and RTs. In this perspective, we expect more efficient individuals to show higher accuracy and relatively low RTs, while less efficient individuals should show the opposite tendency registering low accuracy with low RTs, whereas reflexive individuals would register high accuracy but also higher RTs. We also expect that possibly different profiles of functioning could be differentially associated with self-reported difficulties in ER and behavioral problems, since (i) the ability to use inhibitory abilities plays a role in the regulation of thoughts and feelings (e.g., Rothbart et al., 2007), as individuals need to flexibly attend to environmental stimuli and plan effective coping strategies, and (ii) difficulties with effortful control, including inhibition, have been linked to internalizing problems (e.g., Eisenberg et al., 2001).

To examine profiles of inhibition in adolescence, we administered tasks that test the ability to manage visual interference, to inhibit an automatic response, to stop an ongoing response (in both neutral and emotional conditions), and to inhibit eye movement. These tasks are common and well-established measures of different abilities connected to inhibition (Miyake & Friedman, 2012; Miyake et al., 2000). In addition, we asked participants to complete two self-report measures of difficulties in ER and behavior, to test their association with profile membership. This is a secondary data analysis as the data considered in the present study are part of a larger cross-sectional study aimed to investigate the latent organization of EF and the possible impact on ER in adolescence (see Malagoli 2018a, b).

## Method

### Participants

A convenience sample of 240 (158 females) 14-to-19-year-old high school Italian students participated in this study. Participants were recruited from 33 classes at 5 public schools and belonged to the middle class. The student’s participation in the study was voluntary, recruitment was organized at school, prior to the presentation of the study to the classes. Informed, written consent was obtained from school principals, participants' parents, and the participants themselves before data collection. Participants were excluded if Italian was not their first language or if they had been diagnosed with any disease or neurological/mental disorder. On the basis of the information provided by the school and by the participants throughout the youth self-report (YSR, 11–18 years, Achenbach & Rescorla, 2001), four participants were excluded for Italian not being their first language, eight due to learning disabilities, and one due to neurological issues. The final sample included 227 participants (148 females; mean age:16.9 years, SD: 18.57 months).

### Materials and procedure

A battery of inhibitory tasks was administered during two individual sessions that lasted approximately 45 min each, in a quiet room provided by the school. The participants were also asked to fill two self-reports measuring maladaptive outcomes and ER difficulties.

#### Inhibition tasks

##### ***Flanker task*** (Eriksen & Eriksen, 1974)

This task assesses the ability to manage visual interference and requires a fast response to a centrally presented target stimulus, in this case arrows pointing left or right, flanked by several distractor stimuli (arrows or horizontal bars) that can activate conflict responses. Congruent, incongruent, and neutral trials were shown. The task comprised a practice block of six trials and a test block of 48 trials (16 trials for each condition).

##### ***Go-No Go*** (Donders, 1969)

The Go-No Go is designed to assess the ability to stop an automatic response. The task requires participants to press a button when a given figure target (a blue square) is displayed and to refrain from pressing if any other figure (a blue rectangle) is displayed (20% of the stimuli). Participants practiced on 20 trials and then received 100 target trials. Feedback was provided for every answer. The stimulus duration time was 1500 ms.

##### ***Stop signal task*** (Logan, 1994)

This task aimed to measure response inhibition (Lappin & Eriksen, 1966). It consisted of a practice phase of 32 trials and an experimental phase of three blocks of 64 trials. Each trial started with the presentation of a fixation sign, which was replaced by the primary task (an arrow pointing left or right) stimulus after 250 ms. The stimulus remained on the screen until the participant responded or 1250 ms had elapsed. The default interval between stimuli was fixed to 2000 ms. During “stop” trials, a signal was presented after a variable Stop Signal Delay (SSD). Participants were informed that the signal would be delayed if they slowed down their responses to wait for the signal.

##### ***Antisaccade task*** (adapted from Roberts et al., 1994)

This task is a common measure of oculomotor inhibition. A fixation point appeared in the middle of the computer screen for a variable amount of time. A visual cue (a black square) then appeared on one side of the screen for 175 ms, followed by the target stimulus (an arrow inside of an open square) on the opposite side for 150 ms. The target was then masked until the participant pressed a button to indicate the direction of the target or until 1250 ms had elapsed. Participants practiced on 22 trials and then received 90 target trials.

##### ***Emotional Go-No Go task*** (Hare et al., 2005)

This type of Go-No Go was meant to measure the inhibition of an automatic response in a more emotional condition. The set of stimuli consists of grayscale images of 10 adults (five males; Ekman & Friesen, 1976) showing three different expressions (happy, fearful, and calm/neutral). Face stimuli were presented individually in the center of the screen. Participants were instructed to press the spacebar as fast as they could when the named expression was presented and to refrain from doing so in the “no-go” condition (30% of trials). The test comprised 6 randomized blocks, counterbalanced for each emotion and neutral face with 50 randomized trials for each condition. In each block, only two target emotional stimuli are presented, one in the “go” condition and one in the “no-go” condition. Stimulus duration was 500 ms, with 1000 ms between trials. Practice trials were administered.

Cronbach’s alphas for all these tasks ranged from 0.39 and 0.85 (see Table 1). For all tasks, the measures considered in this study were accuracy and reaction times (RTs). Regarding the Stop signal task, we decided to not use the general index for efficiency, the SSRT (the Stop Signal Reaction Time) which provides the estimation of the covert latency of the stop process and which we did use in a previous study (Malagoli 2018a) meant to investigate the latent components of inhibition and Working Memory (WM) in adolescence. This choice relies on the characteristics of the Stop signal task paradigm that throughout the SSRT offers a more global measure of inhibitory efficiency while throughout the RT measured in the no-stop (go) condition accounts for an increased variability (Matzke, Verbruggen & Logan, 2019) allowing us to take more into account possible differences in the baseline of inhibitory processes.

**Table 1:** Correlations between the executive functions scores and their descriptive statistics and the 16 correlates

	10 Executive Functions Scores																16 Correlates									
	1	2	3	4	5	6	7	8	9	10	11	12	13	14	15	16	17	18	19	20	21	22	23	24	25	26
1. Flanker Accuracy	0.39																									
2. Flanker RT	**0.47**	0.9																								
3. Go-No Go Accuracy	**0.46**	0.12	0.7																							
4. Go-No Go RT	**0.38**	**0.52**	**0.27**	0.77																						
5. No Stop Accuracy	**0.23**	0.05	0.13	− 0.03	0.42																					
6. No Stop RT	**0.3**	*0.13*	**0.27**	0.04	**0.64**	0.98																				
7.AS accuracy	0.04	− 0.12	*0.15*	**− 0.22**	0.11	0.09	0.92																			
8. AS RT	0.1	**0.34**	− 0.05	**0.33**	0.05	0.07	− 0.2	**0.89**																		
9. Emo No Go Accuracy	0.21	− 0.02	**0.48**	− 0.08	**0.25**	**0.34**	**0.23**	− 0.05	0.73																	
10.Emo Go RT	**0.32**	**0.47**	*0.16*	**0.53**	0.02	*0.15*	**− 0.23**	0.18	0.11	0.85																
11. Gender°	− 0.04	0.08	− 0.1	− 0.09	− 0.06	0.06	0.13	− 0.09	− 0.12	0.03	1															
12. Age	*− 0.14*	**− 0.26**	*0.15*	**− 0.22**	− 0.05	− 0.03	0.21	**− 0.27**	**0.24**	− 0.20	0.05	1														
13. YSR-Anxious/Depressed	0.05	0.05	0.06	0.2	0.03	− 0.02	− 0.01	0.03	0.12	0.05	**− 0.32**	0.05	0.74													
14. YSR-Withdrawn/Depressed	− 0.05	*− 0.15*	0.07	0.03	− 0.01	− 0.06	− 0.02	− 0.04	*0.14*	− 0.11	*− 0.15*	**0.23**	**0.64**	0.75												
15. YSR-Somatic Complaints	− 0.04	− 0.03	0.04	0.1	0.01	− 0.06	− 0.03	0.1	0.05	0.03	**− 0.33**	0.09	**0.52**	**0.41**	0.72											
16. YSR—Social Problems	− 0.04	0	− 0.01	0.13	0.03	− 0.1	0	− 0.01	0.05	0	− 0.13	0.04	**0.66**	**0.61**	**0.4**	0.71										
17. YSR-Thought Problems	0.02	0.01	0.03	0.04	0.1	0.05	0.09	0.06	*0.14*	0.04	*− 0.14*	0.09	**0.49**	**0.35**	**0.44**	**0.45**	0.72									
18. YSR-Attention Problems	0.09	0.06	− 0.05	0.09	0.12	0.07	0.11	− 0.03	0.02	0.09	− 0.02	0	**0.4**	**0.27**	**0.34**	**0.45**	**0.45**	0.73								
19. YSR-Rule-Breaking Behavior	0.03	− 0.03	− 0.06	− 0.04	0.09	0.03	0	− 0.01	0.03	− 0.02	*0.14*	*0.16*	0.18	**0.29**	**0.34**	**0.33**	**0.4**	**0.43**	0.73							
20. YSR-Aggressive Behavior	0.08	− 0.02	0.01	− 0.06	*0.15*	0.06	0.06	0.03	0.08	0	− 0.07	0.12	**0.35**	**0.25**	**0.26**	**0.51**	**0.5**	**0.43**	**0.5**	0.7						
21. DERS-Non-acceptance	− 0.04	0.02	− 0.04	0.07	− 0.05	− 0.1	− 0.01	0.06	0.06	− 0.02	− 0.12	0.07	**0.5**	**0.35**	**0.29**	**0.36**	**0.26**	**0.28**	**0.22**	**0.24**	0.79					
22. DERS-Goals	0.12	*0.16*	0.1	0.18	0.13	0.21	0.01	0.07	*0.16*	*0.16*	− 0.08	− 0.03	**0.31**	**0.25**	**0.3**	**0.25**	**0.31**	**0.44**	0.21	**0.22**	**0.39**	0.85				
23. DERS-Impulse	− 0.07	0	− 0.08	0.01	0.03	0.04	0.11	0.01	0.02	− 0.02	*− 0.15*	0.07	**0.39**	**0.29**	**0.25**	**0.4**	**0.4**	**0.4**	**0.23**	**0.47**	**0.48**	**0.47**	0.84			
24. DERS-Awareness	0.07	0.03	*− 0.14*	0.03	− 0.12	− 0.08	− 0.13	0.07	− 0.12	0.12	− 0.05	**− 0.24**	*0.14*	*0.16*	0.06	0.21	0.04	0.18	*0.15*	*0.13*	0.01	0.02	0.08	0.72		
25. DERS-Strategies	− 0.02	*0.14*	0.02	0.18	− 0.01	0.03	− 0.03	0.08	0.04	0.13	*− 0.16*	0	**0.51**	**0.42**	**0.32**	**0.43**	**0.29**	**0.38**	*0.17*	**0.25**	**0.6**	**0.52**	**0.55**	0.12	0.89	
26. DERS-Clarity	0.05	0.03	0.03	0.11	0.01	− 0.03	− 0.13	0.05	0	− 0.06	**− 0.38**	− 0.12	**0.41**	**0.35**	**0.25**	**0.33**	0.2	**0.32**	**0.23**	**0.24**	**0.32**	**0.23**	**0.34**	**0.4**	**0.49**	0.84
% Accuracy	91%		84%		85%		68%		65%																	
Mean	14.59	443.2	16.8	367.57	25.34	625.38	60.84	463	58.22	414.96																
Standard Deviation	1.33	57.52	2.7	45.51	2.01	159.14	14.35	93.6	9.43	37.29																
Minimum	11	306	9	233	18	361	29	183	33	325																
Maximum	16	614	20	504	30	1078	87	705	76	525																

*N* = 227; °: coded as Females = 0, Males = 1; *AS* Antisaccade, *Emo* Emotional Go-No Go, *YSR* Youth Self report, *DERS* Difficulties in Emotion Regulation Scale, Bolded coefficients are significant at *p* < .001; Underlined coefficients are significant at *p* < .01; Italicized coefficients are significant at *p* < .05. C ronbach’s alphas are shown on the main diagonal

#### Self-report measures

##### Youth self-report (YSR, 11–18 years, Achenbach & Rescorla, 2001

Italian version as available on the http://www.aseba.org website). The YSR is part of the Achenbach System of Empirically Based Assessments (ASEBA). It provides an assessment of the respondent’s social and emotional functioning. The 2001 revised YSR comprises 112 problem items and provides scores in eight subscales (Anxious/Depressed, Withdrawn/Depressed, Somatic Complaints, Social Problems, Thought Problems, Attention Problems, Rule-Breaking Behavior, and Aggressive Behavior). Cronbach's alphas were larger than 0.70 for all scales, and are reported in Table 1.

##### Difficulties in Emotion Regulation Scale (DERS, Gratz & Roemer, 2004

Italian version in Giromini et al., 2012)*.* The DERS is a 36-item, self-report measure developed to assess clinically relevant difficulties in ER. Items provide scores on six scales (Nonacceptance, Goals, Impulse; Awareness; Strategies; Clarity).

Cronbach’s alphas were computed for the total DERS score and for each subscale, and were all larger than 0.70 (Table 1). More details about the method and the instruments are provided in Sect. 1 of the Supplementary Materials (SM).

### Statistical Analysis

Descriptive statistics and zero-order (Pearson) correlations among measures were computed. Outlier values (i.e., more than three standard deviations from the mean) were excluded. Total excluded values represented 0.62% of the data.

We then used latent profile analysis (LPA) to identify groups of individuals with qualitatively (profile shape) and/or quantitatively (profile level) distinct patterns on EF tasks, and to investigate the association of group membership with a diverse set of correlates (gender, age, behavioral, and emotional problems, and difficulties in ER). LPA is a person-centered approach that focuses on relations among individuals to sort them into groups in which they are similar to each other and different from those in other groups (Lubke & Muthén, 2005; Pastor et al., 2007). More details on this method are provided in Sect. 2 of the Supplementary Materials (SM).

LPAs were conducted with Mplus (version 7.0; Muthén & Muthén, 2012) using the TYPE = MIXTURE COMPLEX option to take into account the nesting of participants into classrooms. In subsequent analyses, we included the set of correlates using the 3-step auxiliary variable approach in Mplus (Asparouhov & Muthén, 2014). Following this approach, in the first step a latent profile model is estimated using only latent profile indicator variables. In the second step, the most likely class variable is created using the latent class posterior distribution obtained during the first step. In the third step, the most likely class is regressed on the predictor variables, taking into account the misclassification in the second step (Asparouhov & Muthén, 2014). Missing values were handled using the Full Information Maximum Likelihood procedure implemented in Mplus 7.0.

## Results

Descriptive statistics and correlations are reported in Table 1. With regard to DERS scores, considering all the 6 subscales, none of the participants presented scores above the score that is considered the threshold for clinical risk for all the 6 subscales and only 1.3% of participants presented scores above the threshold for one to two scales. Regarding YSR, considering all 8 subscales, 48% of participants scored above the threshold in at least one scale, none of the participants reported scores above the threshold in all the 8 scales and only 6.6% of participants reported scores above the threshold for 4 or more subscales.

Performance in inhibition tasks did not differ between genders (see correlations with gender in Table 1 and Sect. 4 of the Supplementary Materials); age was significantly associated, albeit weakly (*r*s <|.30|), with slower RTs and higher accuracy. The inhibitory tasks were associated with each other, consistent with previous findings. Significant correlations were also found for self-report measures, while only a few weak correlations were found between EF and self-report measures (|.18|< rs <|.27|). Notably, the RTs on the Go-No Go task were positively associated with YSR-Anxious/Depressed, DERS-Difficulties in Engaging in Goal Directed Behavior, and DERS-Limited Access to ER Strategies. DERS-Difficulties in Engaging in Goal Directed Behavior were also positively correlated with RTs on the Stop signal task.

**Fig. 1:**
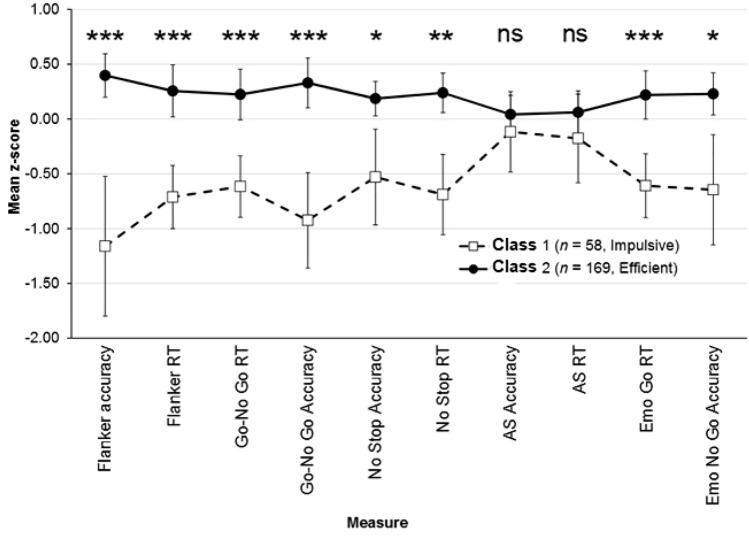
Profiles of EF task scores based on latent profiles. *RT* reaction time, *AS* Antisaccade, Emo Emotional Go-No Go; *** *p* < .001; **: *p* < .01; *: *p* < .05

Results of the LPA are reported in Table 2. For the three information indices (AIC, BIC, and SSA–BIC), the values continued to decrease across the range of models considered, apparently suggesting that we should consider at least eight groups. None of the models resulted in groups with less than 1% of the cases, whereas models positing more than five groups each resulted in at least one group with less than 5% of the cases. The results based on the significance tests (see SM) were very similar to each other, and they converged in suggesting that the appropriate number of groups was two (Class 1 size: 58; Class 2 size: 169). We thus used the Mplus MODEL CONSTRAINT option to specify class mean score comparisons for each of the measures used to perform the LPA. After adjustment for multiple comparisons performed with the adaptive Benjamini and Hochberg (2000) step-up false discovery rate-controlling procedure, all comparisons revealed that participants in the larger class obtained significantly higher scores than their counterparts in the smaller class in all tasks, except in the Antisaccade task (Fig. 1). Class 1 participants showed a general impulsive tendency in all tasks, and this was reflected in a worse performance in terms of accuracy. For this reason, we labeled it “Impulsive”. Conversely, Class 2 participants showed a good performance on the majority of tasks, with high accuracy scores and quite low RTs. We therefore labeled it “Reflexive”. When we used the 3-step method for latent class predictor variables, we found that none of the predictors was significantly associated with class membership, with very small odds ratios (Table 3). Evidence of the replicability of the obtained classes was obtained by drawing 500 random samples of 170 participants (~ 75% of the original sample) and performing the same analyses of the manuscript on each of them. The details of the analyses and the results are reported in the supplementary materials.

**Table 2:** Goodness of fit for Latent Profile Models Based on Different Numbers of Groups

No Groups	No Parm	AIC	BIC	SSA-BIC	p LMR (BLRT)	Smallest Group Size (Percentage)	Entropy
1	20	6301.505	6370.004	6306.618	–	–	–
2	31	6096.028	6202.202	6103.954	.0328 (.0348)	58 (26%)	0.808
3	42	5946.999	6090.847	5957.737	.1222 (.1258)	60 (26%)	0.818
4	53	5867.885	6049.407	5881.435	.2965 (.3018)	40 (18%)	0.819
5	64	5830.078	6019.275	5846.440	.6087 (.6125)	13 (6%)	0.853
6	75	5808.529	5995.401	5827.705	.5305 (.5325)	7 (3%)	0.865
7	86	5784.160	5973.706	5806.147	.6677 (.6687)	10 (4%)	0.863
8	97	5772.040	5962.260	5796.840	.6211 (.6221)	7 (3%)	0.876

*No* number, *Parm* parameters, *AIC* Akaike’s Information Criterion, *BIC* Bayesian Information Criterion, *SSA–BIC* sample-size adjusted Bayesian Information Criterion, *p LMR (BLRT)* p values for the Lo–Mendell–Rubin likelihood and the bootstrap likelihood ratio test for *K* versus *K*–1 classes. *N* = 227

**Table 3:** Association of the two latent classes (reference: class 2) to the correlates (auxiliary) variables

Predictor	Estimate	SE	*p*
Age	0.26	0.27	.336
Gender	− 0.26	0.57	.641
YSR—Anxious/Depressed	− 0.15	0.08	.055
YSR—Withdrawn/Depressed	0.08	0.16	.632
YSR—Somatic Complaints	0.04	0.10	.650
YSR—Social Problems	0.17	0.19	.393
YSR—Thought Problems	− 0.10	0.10	.302
YSR—Attention Problems	− 0.06	0.11	.622
YSR—Rule-Breaking Behavior	− 0.05	0.09	.560
YSR—Aggressive Behavior	− 0.04	0.10	.692
DERS—Non-acceptance	0.36	0.33	.276
DERS—Goals	− 0.72	0.59	.225
DERS—Impulse	0.66	0.43	.127
DERS—Awareness	− 0.11	0.33	.738
DERS—Strategies	− 0.22	0.41	.584
DERS—Clarity	0.12	0.43	.785

*SE* standard error, *YSR* Youth Self Report, *DERS* Difficulties in Emotion Regulation Scale. P-values are not corrected for multiple comparisons. When the Benjamini–Hochberg procedure was applied, the significance level of all the estimates was .784

## Discussion

In this study, we investigated whether different profiles in scores on inhibition tasks exist and whether they are associated with difficulties in ER and adaptive behavior skills. The results showed that inhibition tasks are associated with each other, suggesting that these abilities are actually tightly connected (Friedman & Miyake, 2004). We did not find gender differences in inhibition tasks performance, while the association with age, with older adolescents showing slower RTs and higher accuracy, is consistent with previous studies and indicates a development in inhibitory abilities during adolescence (e.g., Diamond, 2013; Nigg, 2000). Scores on self-report measures of ER and emotional/behavioral problems were also significantly associated. This result is consistent with the findings presented by Kaufman et al. (2015) who found that scores on the DERS were significantly associated with scores on the YSR, and in general with the existing literature that addresses how emotion (dys)regulation indicators may be considered an indicator for vulnerabilities across diagnoses (e.g., Beauchaine & Thayer, 2015).

We found weak but significant positive bivariate associations of RTs to inhibitory tasks, such as the Go-No Go and the Stop signal Task, with measures of anxiety/depression, difficulties in engaging in goal-directed behavior, and limited access to ER strategies. This result is consistent with the previous results (Lopez-Vergara & Colder, 2013), as individual differences in reaction to aversive stimuli can lead to dysregulated impulsive behavior and individuals who are highly sensitive to punishment, in fact, seem to have an attentional bias to certain emotional cues (Wallace et al., 1991).

The main aim of this paper was to investigate whether individual profiles of inhibitory efficiency, assessed throughout a specific selection of inhibitory tasks accounting, could be distinguished. Using LPA, we found two different profiles of inhibitory control abilities that differed in terms of accuracy and RTs on EF tasks. Participants in the latent class labeled as Impulsive showed a general tendency to respond fast in all tasks, with worse performances in terms of accuracy. Reflexive participants showed a good inhibitory control performance both in terms of speed and accuracy, with a good performance on the majority of tasks, with relatively high accuracy scores and relatively low RTs. In everyday life, being reflexive means being able to process stimuli at a speed that also allows for accuracy. These results are consistent with the limited existing literature about differences in profiling of inhibition development that suggest the existence of particularities at this level (Schiller et al., 2014) and indicate that it is actually possible to identify profiles of functioning that differ in inhibitory efficiency (Haghighi et al., 2015; Quiroga, et al., 2007).

No differences in the performance on the Antisaccade task were found between the two classes. As a possible explanation, it should be noted that this task involves the automatic control of eye movements. In fact, the Antisaccade task requires at least two processes: the suppression of a reflexive prosaccade toward the stimulus and the generation of the antisaccade away from the stimulus to an empty location, that involve oculomotor processes (Nigg, 2000). It is unclear whether the same brain systems involved in the inhibition of eye movements are also engaged in the inhibition of other behaviors. These characteristics may explain the lack of difference in performance between the two classes on this specific task.

It is important to point out that the classes we identified in this study reflect different profiles that participants exhibited in the specific inhibitory tasks we chose for the study. In other words, this may not reflect impulsiveness or reflectiveness in other general domains, as the goal of the study was a more precise investigation about individual differences in inhibitory control on a cognitive level. Nevertheless, to the best of our knowledge, this is the first study showing that different profiles of inhibitory efficiency can be distinguished in adolescents.

The results of this study add to the existing literature as they show that it is actually possible to distinguish different profiles in the cognitive ability to inhibit responses, as these baselines in individual differences may be proper characteristics of individuals, even in adolescence. Studies with adults have already shown the existence of different characteristics in terms of impulsivity *vs* reflexivity (Giedd, 2010; Giedd et al., 2008) in the general population, but what was missing was an investigation on previous stages of development. The results of this study support this evidence and add knowledge about a less investigated stage in development**.**

When we regressed class membership on gender, age, and scores on measures of ER and behavioral difficulties, we found no significant results. This outcome did not support our hypothesis that different styles of inhibition correspond to more difficulties or at least to a weakness in these domains, indeed bivariate correlations indicated that performance on inhibitory tasks and scores on self-report measures were partially (weakly) correlated. A similar pattern of results also emerged in a study by Romer et al. (2009) that analyzed EF and impulsivity as correlates of risk taking and problem behavior in preadolescents. In this study, too, YSR scores showed no relation with more cognitive aspects. The specificity of the abilities considered in the study should be taken into consideration. Some aspects of behavioral impulsivity may be instantly realized in everyday life, since individuals can experience the immediate consequences of them, while emotions may need the ability to mentalize feelings through deep introspection to be fully understood. As mentioned above, this ability in adolescence is still developing (Keulers et al., 2010). In fact, while computerized tasks are direct instruments, to accurately answer self-reports (especially those concerning ER), participants must possess self-awareness about existing difficulties, together with being able to evocate specific situations. Interestingly, the most recent literature on the topic suggested that questionnaire measures of self-control and performance measures of inhibition and related EF are largely unrelated to each other (Paap et al., 2020; Saunders, Milyavskaya, Etz, Randles & Inzlicht, 2020). However, Sanders et al., (2020) made clear that they do not claim that their results would invalidate one measure or the other. Rather, they argue that their results suggest that inhibitory tasks, such as the Stroop and Flanker tasks, “*do not reflect the broader individual difference construct that is reflected in self-report scales, and, equally, that scores on the self-control scale are not analogous to the processes assessed by the Stroop and flanker tasks”* (p. 11).

More generally, previous studies reported low correlations between direct and indirect measures of EF (Toplak et al., 2013) and even EF training studies report that EF improvements struggle to generalize to other cognitive and behavioral domains (Diamond & Ling, 2016). In general, studies that attempted to measure these components using direct and indirect measures reported nonsignificant correlations between inhibition tasks and self-report measures of impulsivity (Eisenberg et al., 2019; Nęcka et al., 2018; Stahl et al., 2013) and conscientiousness (Fleming et al., 2016).

It should be considered that diverse behavioral outcomes may be associated with different EF components. In this regard, previous studies considering typical and atypical development showed that in understanding the relation between these cognitive abilities and functional profiles, a distinction should be considered between the cool and hot aspects of EF (Campbell & Von Stauffenberg, 2009). Whereas cool EF is more likely to be involved in relatively abstract, decontextualized problems, hot EF tasks require the regulation of affect and motivation (Zelazo & Müller, 2002). This latter aspect, namely, affective control, can be more associated with ER than cool measures of inhibition (Schweizer et al., 2020). In the current study, mainly performances on cool inhibition tasks were examined, which may be less associated with social and emotional outcomes than hot EF task performance.

A different perspective on this result may also rely on an adaptive role that the ability to react fast may play in certain cases (e.g., running away before getting into a fight). In these cases, being fast could be more important than being accurate. The YRS and the DERS questionnaires indirectly investigate, respectively, social aspects of regulation, like the tendency to “lose control”/ “get into a fight”/break the rules”, and emotional aspects of regulation, as in the case of DERS-Impulse scale, assessing difficulties in controlling one's behavior “*when feeling upse*t” which are specific social aspects at one extreme of impulsivity. However, being impulsive may not be necessarily linked to extreme behaviors, since it may be modulated as an inner “rhythm” of the individual. In this perspective, being impulsive may not be a risk factor per se. It may depend on the task/situation, so that being impulsive may not lead to extreme outcomes but simply be a baseline, which needs to be really pushed to the extreme and co-occur with other factors to become a risk factor (Soloff et al., 2010; Vitaro et al., 1998).

A few limitations of this study warrant mention. First, since we used convenience sampling, our sample cannot be considered fully representative of the adolescent population. Consequently, the results might be of limited generalizability, e.g., because of the noise due to the sociodemographic variation that cannot be controlled (see e.g., Bornstein et al., 2013). Convenience sampling is known to provide insufficient power to detect differences among sociodemographic subgroups, and this might be another reason why we did not find statistically significant associations of profile membership with gender and age. Second, since correlations between test scores are attenuated by different methods other than measurement error, the lack of association of profile membership with measures of ER and behavioral difficulties could also be explained by a lack of power. Future studies are thus invited to enrol larger and more representative samples, possibly also involving clinical samples, in order to address and investigate further these issues while also considering noncluster-based methods to approach similar data.

The third limitation is the relatively low reliability of accuracy scores for the Flanker and the Stop signal tasks, but this is an issue often reported for executive tasks (Denckla, 1996; Miyake et al., 2000), especially for inhibition ones (Friedman & Miyake, 2004). Finally, the two profiles were not validated against a noncomputerized task, to investigate whether the use of a computerized task could bias the results of the LPA. Further research is thus needed to shed light on this issue. A fourth limitation is that in the present study adaptive behavioral skills have not been investigated throughout ecological and direct measures, yet only assessed throughout self-report measures. Future studies are; thus, invited to include also specific measures to explore deeper this topic.

Despite these limitations, the study contributes in adding knowledge about the existence of individual differences considering the trajectory of a core cognitive ability as inhibition in a challenging developmental period. The presented results also have important practical implications by suggesting that the impulsive and reflexive profiles are not necessarily associated with maladaptive behaviors or ER problems when the continuum of individual differences is considered. Therefore, extreme profiles showing behavioral and emotional problems could be considered qualitatively different from the intermediate profiles, such as those found in the present study (see, e.g., Kagan, 2013).

The present results also suggest important practical implications for teaching and in general for the educational fieldwork. Accounting for individual differences explicitly working towards the strengthening of inhibitory related skills (e.g., structuring differently the learning environment, challenging peer education) or proactively supporting students in the application of strategic learning behaviors or activities, may have a crucial role in fostering students' ability to be efficient in inhibitory related skills while becoming more strategic and proficient in learning.

## Conclusions

The results of this study suggest the existence of specific individual differences in terms of inhibitory efficiency, as two distinct classes that show different profiles in inhibition functioning could be identified: a reflexive and an impulsive one. Class membership was not related to measures of ER or behavioral difficulties, gender, or age, suggesting that, possibly and in line with the most recent literature on the topic, measures of inhibitory control and self-reported measures of self-control and/or impulsivity and consequently appear to be measuring different constructs.

## Supplementary Information

Below is the link to the electronic supplementary material.

Supplementary file1 (DOCX 56 kb)
