# Supplementary material for: Inhibition and individual differences in behavior and emotional regulation in adolescence
Source: Psychol Res. 2021 Aug 7;86(4):1132–44. doi: 10.1007/s00426-021-01565-8 (PMC9090849; doi:10.1007/s00426-021-01565-8)
Supplement: Supplementary file 1 — Supplementary file1 (DOCX 56 kb) [file 426_2021_1565_MOESM1_ESM.docx]

**Supplementary materials for the paper " Inhibition and individual differences in behavior and emotional regulation in adolescence"**

1. **Extended Description of Materials**

***Flanker task*** (Eriksen & Eriksen, 1974). This task assesses the ability to manage visual interference and requires a fast response to a centrally presented target stimulus that is flanked by several distractor stimuli that can activate conflict responses. In this study we used arrows (0.32 cm) pointing left or right. Congruent, incongruent, and neutral trials were shown. The task comprised a practice block of six trials and a test block of 48 trials (16 trials for each condition).

***Go-No Go*** (Donders, 1969). The Go-No Go is a classical inhibition paradigm meant to assess the ability to stop an automatic response. The task requires participants to press a button when a given figure target (a blue square) is displayed and to refrain from pressing if any other figure is displayed (20% of the stimuli). The task consists of a practice phase of 20 trials and a test phase of 100 trials. Participants are asked to answer as fast as they can every time the Go stimulus appears. Feedback is received every time they press. The stimulus duration time is 1500 ms.

***Stop Signal task*** (Logan, 1994). This task aimed to measure response inhibition (Lappin & Eriksen, 1966). The stop signal task consists of a practice phase of 32 trials and an experimental phase of three blocks of 64 trials. In both phases, each trial starts with the presentation of a fixation sign, which is replaced by the primary-task stimulus after 250 ms. By default, the response keys are “D” (for left arrow) and “K” (for right arrow). The stimulus remains on the screen until the participant responds or 1250 ms (i.e., the maximal RT) have elapsed. The default interval between stimuli is fixed to 2000 ms for all trials. During stop-signal trials, a stop signal is presented after a variable Stop Signal Delay (SSD). SSD is initially set at 250 ms and adjusted continuously with the staircase procedure: if the response inhibition is successful, SSD increases by 50 ms; if it is unsuccessful, SSD decreases by 50 ms. Response registration continued during stop-signal presentation. Participants were informed that the signal will be delayed if they slow down their responses to wait for the stop signal.

***Antisaccade task*** (adapted from Roberts, Hager, & Heron, 1994). This task is a common measure of oculomotor inhibition. A fixation point appears in the middle of the computer screen for a variable amount of time (one of nine times between 1500 and 3500 ms in 250-ms intervals). A visual cue (0.32 cm) in the shape of a black square then appears on one side of the screen for 175 ms, followed by the target stimulus (an arrow inside of an open 1.59 cm square) on the opposite side for 150 ms. The target is then masked with a gray square that remains on the screen until the participant presses a button to indicate the direction of the arrow (left, up, or right) or until 1250 ms (i.e., the maximal RT) have elapsed. Cues and the targets are presented 8.64 cm from the fixation point (on opposite sides). Participants practice on 22 trials and then receive 90 target trials.

### *Emotional trial*

***Emotional Go-No Go task*** (Hare, Tottenham, Davidson, Glover, & Casey, 2005). This particular type of Go-No Go is meant to measure the inhibition of an automatic response in a more emotional condition, created using faces that express a specific emotion. The set of stimuli consists of grayscale images of 10 adults (five males and five females; Ekman & Friesen, 1976) showing three different expressions (happy, fearful, and neutral). The visual angle is approximately 12°. The task requires participants to press the space bar when a certain facial expression target (e.g., neutral) is displayed. Face stimuli are presented individually in the center of the screen. Participants are instructed to press the space bar as fast as they can when the named expression is presented. They are also asked to refrain from pressing the space bar when a “no-go” facial expression stimulus (e.g., fearful face) appears, and these “no-go” trials occur infrequently (30% of the trials). They are not told which faces are “no-go”, but are instructed to refrain from pressing for “any face other than the ‘go’ expression.” In each block, an emotional expression (happy, fearful, or sad) is always paired with a neutral expression, and, depending on the block, either the emotional expression serves as the “go” stimulus (when neutral is the “no-go” stimulus) or as the “no-go” stimulus (when neutral is the “go” stimulus). The test is composed by 6 randomized blocks of “Go-No Go” pairs, counterbalanced for each emotion and neutral face with 50 randomized trials for each condition. Stimulus duration is 500 ms with 1000 ms between trials to ensure that participants have enough time to respond. Practice trials are administered to ensure that participants have understood the task.

### *Self-report measures*

***Youth Self-Report*** (YSR*,* 11-18 years*,* Achenbach & Rescorla, 2001, Italian version as available on the http://www.aseba.org website). This questionnaire is one of a family of screening tools for behavioral and emotional problems in children and adolescents. The YSR is part of the Achenbach System of Empirically Based Assessments (ASEBA). It provides an assessment of the respondent’s social and emotional functioning. The 2001 revised YSR comprises 112 problem items in a six-month time period. These 112 items are scored using a three-point Likert-type frequency scale (0=absent, 1= occurs sometimes, 2=occurs often), and provide scores in eight subscales: Anxious/Depressed, Withdrawn/Depressed, Somatic Complaints, Social Problems, Thought Problems, Attention Problems, Rule-Breaking Behavior, and Aggressive Behavior. Some subscales are clustered in order to identify individual’s externalizing or internalizing tendencies: Internalizing is the resulting profile from Anxious/ Depressed, Withdrawn/Depressed, and Somatic Complaints scores; while Rule-Breaking Behavior and Aggressive Behavior make up the Externalizing profile.

***Difficulties in Emotion Regulation Scale*** (DERS, Gratz & Roemer, 2004; Italian version in Giromini, Velotti, de Campora, Bonalume, & Zavattini, 2012)*.* The DERS is a 36 item self-report measure developed to assess clinically relevant difficulties in emotion regulation. Items provide scores on six scales: Non-acceptance of Emotional Responses (Non-acceptance, 6 items); Difficulties Engaging in Goal-Directed Behavior (Goals, 5 items); Impulse Control Difficulties (Impulse, 6 items); Lack of Emotional Awareness (Awareness, 6 items); Limited Access to Emotion Regulation Strategies (Strategies, 8 items); and Lack of Emotional Clarity (Clarity, 5 items). Participants are asked to indicate how often each of the 36 items applies to them on a 5-point Likert-type frequency scale ranging from 1 (*almost never*) to 5 (*almost always*). To determine the internal consistency of the DERS items, Cronbach’s alphas were calculated for the total DERS score and for each of the six subscales.

1. **Description of Latent Profile Analysis**

Latent Profile Analysis is a person-centered approach that focuses on relations among individuals in order to sort them into groups in which they are similar to each other and different from those in other groups (Lubke & Muthén, 2005; Pastor, Barron, Miller, & Davis, 2007).

LPA decomposes the covariances among *individuals* in order to identify clusters of individuals (Bauer & Curran, 2004). It is assumed that the dataset consists of unobservable, heterogeneous subgroups of individuals with different probability distributions. LPA is a model-based procedure that allows for a more flexible model specification than cluster analysis, which is essentially an exploratory technique and, to some respects, a very restricted specification of a latent profile model. LPA offers several advantages over cluster analysis techniques (for a discussion, see Pastor et al., 2007), such as modelling the association of class membership with external covariates in the same model.

LPA also has the advantage of providing fit indices that make it possible to compare different models and make informed decisions regarding the optimal number of underlying classes. In order to determine this, we evaluated models positing between 1 and 8 groups in relation to five commonly used indices of fit (Nylund, Asparouhov, & Muthén, 2007; Tein, Coxe, & Cham, 2013). We considered information criterion indices, namely, the Bayesian Information Criterion (BIC), the Akaike’s Information Criterion (e.g., Henson, Reise, & Kim, 2007), and the sample-size-adjusted BIC (SSA–BIC; Yang, 2006). More recently, it has been suggested to base the selection of the number of groups on the difference in the log-likelihood ratio tests (Nylund et al., 2007; Tein et al., 2013). These tests are equivalent to chi-square difference tests for nested structural equation models, for which a significant difference is considered as support for using a more complex model rather than a more parsimonious one. Although the difference in the log-likelihood ratio tests (LRT) for two such models does not have a chi-square distribution, the fit of the two models can be compared with a test developed by Lo, Mendell, and Rubin (LMR; 2001) and its bootstrapped version (BLRT; McLachlan & Peel, 2001). Evidence has suggested that LMR and BLRT tests of significance are very effective in determining the appropriate number of groups (Nylund et al., 2007; Tein et al., 2013; Tofighi & Enders, 2007).

1. **Replicability of the Latent Profile Analysis results**

The LPA analyses were performed multiple times with different numbers of initial stage starts and of final stage optimizations (highest number: 1,200 and 200, respectively), and the best loglikelihood was always replicated. Additionally, we sought to replicate the results by drawing 500 random samples of 170 participants (~75% of the original sample) and performing the same analyses of the manuscript on each of them. We report in Table SM1 the quantiles of the distributions of relevant statistics.

Table SM1 Class size (proportions reported in the manuscript: Impulsive: .26; Efficient: .74)

| Class | Min | P2.5 | P10 | P25 | P50 | P75 | P90 | P97.5 | Max |
| --- | --- | --- | --- | --- | --- | --- | --- | --- | --- |
| Impulsive | .13 | .19 | .23 | .25 | .29 | .36 | .72 | .75 | .78 |
| Efficient | .22 | .25 | .28 | .64 | .71 | .75 | .77 | .81 | .87 |

As shown in Table SM1, the median values are close to those actually found on the whole sample, and so are the values that bound the central 50% of the distributions.

We computed for each participant the proportion of times they have been included in the same class as in the original analysis. The results are reported in Table SM2 and show that, especially for the Impulsive class, the central 50% of the distribution included 1.00.

Table SM2 Probability for being classified in the same latent class as in the original analyses

| Class | P0 | P2.5 | P10 | P25 | P50 | P75 | P90 | P97.5 | P100 |
| --- | --- | --- | --- | --- | --- | --- | --- | --- | --- |
| Impulsive | .64 | .78 | .88 | .96 | 1.00 | 1.00 | 1.00 | 1.00 | 1.00 |
| Efficient | .24 | .41 | .69 | .81 | .83 | 1.00 | 1.00 | 1.00 | 1.00 |

We then plotted the profiles resulting from the resampling analysis (Figure SM1). Note that the dots represent the median of the distribution of the 500 model-estimated means, and that the whiskers are bounded by the 2.5th and 97.5th percentiles of such distribution. The pattern is quite similar to the actual Figure 1 included in the original submission.


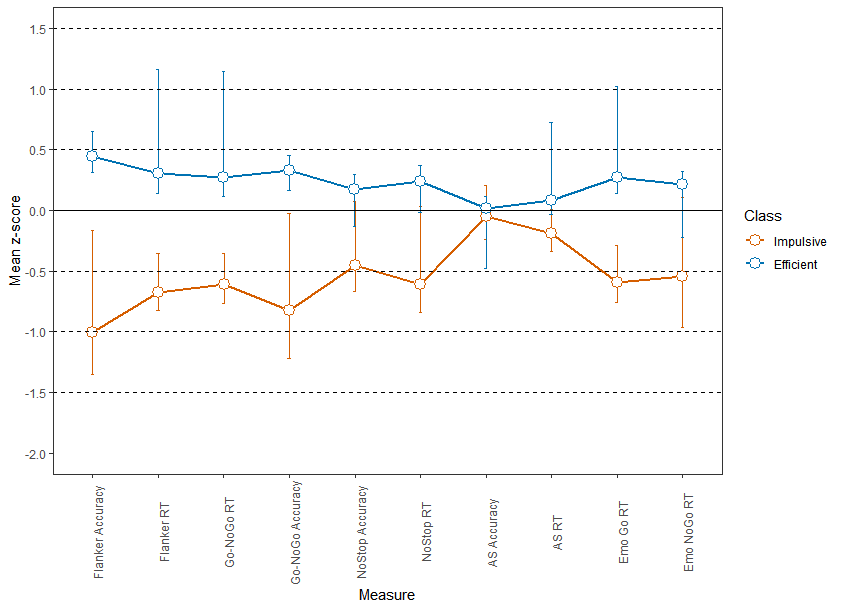


Figure SM1 Profile plot using data from the resampling analyses

Finally, we computed the proportion of times each predictor of class membership turned out to be statistically significant (Table SM3). As the highest proportion was .26, these results suggest that it is unlikely that there is some significant association with class membership.

Table R3 Proportion of times each predictor of class membership was statistically significant

| Predictor | P |
| --- | --- |
| Age | .19 |
| Gender | .21 |
| YSR - Anxious/Depressed | .01 |
| YSR - Withdrawn/Depressed | .01 |
| YSR - Somatic Complaints | .01 |
| YSR - Social Problems | .12 |
| YSR - Thought Problems | .26 |
| YSR - Attention Problems | .01 |
| YSR - Rule-Breaking Behavior | .01 |
| YSR - Aggressive Behavior | .04 |
| DERS - Non-acceptance | .01 |
| DERS – Goals | .04 |
| DERS - Impulse | .00 |
| DERS - Awareness | .01 |
| DERS - Strategies | .03 |
| DERS - Clarity | .10 |

We also computed the bivariate multilevel correlations of class membership with the potential predictors, and we did not find any significant effect (Table SM4).

Table SM4 Bivariate associations of class membership with predictors (r metric). Reference class: Efficient

| Predictor | r | 95% CI |
| --- | --- | --- |
| Age | -.03 | [-0.16; 0.10] |
| Gender | .02 | [-0.11; 0.15] |
| YSR - Anxious/Depressed | -.09 | [-0.22; 0.04] |
| YSR - Withdrawn/Depressed | .00 | [-0.13; 0.13] |
| YSR - Somatic Complaints | .00 | [-0.13; 0.13] |
| YSR - Social Problems | -.03 | [-0.16; 0.10] |
| YSR - Thought Problems | -.10 | [-0.23; 0.03] |
| YSR - Attention Problems | -.10 | [-0.22; 0.04] |
| YSR - Rule-Breaking Behavior | -.05 | [-0.18; 0.08] |
| YSR - Aggressive Behavior | -.06 | [-0.19; 0.07] |
| DERS - Non-acceptance | -.01 | [-0.14; 0.12] |
| DERS - Goals | -.11 | [-0.24; 0.02] |
| DERS - Impulse | .03 | [-0.10; 0.16] |
| DERS - Awareness | .00 | [-0.13; 0.13] |
| DERS - Strategies | -.04 | [-0.17; 0.09] |
| DERS - Clarity | -.01 | [-0.14; 0.12] |

In some cases, the effect size of the coefficient suggested a weak association (*r* >= |.10|) in the direction of the hypotheses. This weak association might be the result of the known attenuation issues due to the partial unreliability of predictors, to class membership being a dichotomous variable (see, e.g., Nunnally, 1975), to correlating measures of different kind (i.e., typical performance with maximum performance measures, which cannot share method variance, Campbell & Fiske, 1955), and to the effect of situational factors that occur in the translation from behavior to results in maximum performance tests (i.e., the *criterion problem*, see Austin & Villanova, 1992).

Summarizing, the lack of significant associations of class membership with the hypothesized predictors can be due to a variety of factors (included the most obvious, namely, that the association is absent or of negligible importance in the population implied by our sample, or that the study was underpowered), and not necessarily to a lack of validity of the results of the LPA, as the simulation study we performed provided evidence of their replicability.

**Additional References**

Austin, J. T., & Villanova, P. (1992). The criterion problem: 1917-1992. *Journal of Applied Psychology, 77*(6), 836–874. doi:10.1037/0021-9010.77.6.836

Bauer, D. J., & Curran, P. J. (2004). The integration of continuous and discrete latent variable models: Potential problems and promising opportunities. *Psychological Methods*, *9*(1), 3-29. doi:10.1037/1082-989X.9.1.3

Campbell, D. T., & Fiske, D. W. (1959). Convergent and discriminant validation by the multitrait-multimethod matrix. *Psychological Bulletin, 56*(2), 81–105. doi:10.1037/h0046016

Henson, J. M., Reise, S. P., & Kim, K. H. (2007). Detecting mixtures from structural model differences using latent variable mixture modeling: A comparison of relative model fit statistics. *Structural Equation Modeling*, *14*(2), 202–226. doi:/10.1080/10705510709336744

Lo, Y., Mendell, N. R., & Rubin, D. B. (2001). Testing the number of components in a normal mixture. *Biometrika*, *88*(3), 767–778. doi:10.1093/biomet/88.3.767

McLachlan, G., & Peel, D. (2001). *Finite mixture models*. New York: Wiley.

Nunnally, J. C. (1975). *Introduction to statistics for psychology and education*. New York: McGraw-Hill.

Nylund, K. L., Asparouhov, T., & Muthén, B. O. (2007). Deciding on the number of classes in latent class analysis and growth mixture modeling: A Monte Carlo simulation study. *Structural Equation Modeling*, *14*(4), 535–569. doi:10.1080/10705510701575396

Tein, J.-Y., Coxe, S., & Cham, H. (2013). Statistical power to detect the correct number of classes in latent profile analysis. *Structural Equation Modeling, 20*(4), 640–657. doi:10.1080/10705511.2013.824781

Tofighi, D., & Enders, C.K. (2007). Identifying the correct number of classes in a growth mixture model. In G. R. Hancock (Ed.), *Advances in Latent Variable Mixture Models* (pp. 317-341), Greenwich: Information Age.

Yang, C.-C. (2006). Evaluating latent class analysis models in qualitative phenotype identification. *Computational Statistics & Data Analysis*, *50*(4), 1090–1104. doi:10.1016/j.csda.2004.11.004

1. **Gender differences in executive functions and personality measures computed as mean differences**

|  | Females | | |  | Males | | |  |  |  |  |  |
| --- | --- | --- | --- | --- | --- | --- | --- | --- | --- | --- | --- | --- |
|  | n | M | SD |  | n | M | SD | t | df | p | p-adj | d |
| *Executive Functions measures* |  |  |  |  |  |  |  |  |  |  |  |  |
| Flanker Accuracy | 147 | 14.63 | 1.32 |  | 76 | 14.57 | 1.37 | 0.32 | 221 | .752 | .752 | 0.04 [-0.28, 0.37] |
| Flanker RT | 147 | 439.97 | 56.58 |  | 79 | 449.46 | 59.14 | -1.18 | 224 | .240 | .350 | -0.17 [-0.49, 0.16] |
| Go-No Go Accuracy | 147 | 370.44 | 46.67 |  | 78 | 362.12 | 43.09 | 1.30 | 223 | .195 | .312 | 0.18 [-0.14, 0.51] |
| Go-No Go RT | 147 | 16.99 | 2.59 |  | 78 | 16.45 | 2.89 | 1.42 | 223 | .156 | .268 | 0.20 [-0.12, 0.52] |
| No Stop Accuracy | 145 | 25.42 | 1.89 |  | 78 | 25.19 | 2.23 | 0.81 | 221 | .419 | .503 | 0.11 [-0.21, 0.44] |
| No Stop RT | 146 | 618.68 | 149.58 |  | 79 | 637.70 | 175.81 | -0.85 | 223 | .396 | .500 | -0.12 [-0.44, 0.21] |
| AS accuracy | 146 | 59.42 | 14.49 |  | 78 | 63.51 | 13.77 | -2.04 | 222 | .043 | .136 | -0.29 [-0.61, 0.04] |
| AS RT | 147 | 469.67 | 94.02 |  | 78 | 450.47 | 92.25 | 1.46 | 223 | .145 | .268 | 0.21 [-0.12, 0.53] |
| Emo No Go Accuracy | 140 | 59.02 | 9.13 |  | 76 | 56.74 | 9.85 | 1.70 | 214 | .091 | .199 | 0.24 [-0.09, 0.57] |
| Emo Go RT | 138 | 414.10 | 37.97 |  | 76 | 416.47 | 36.12 | -0.44 | 212 | .659 | .687 | -0.06 [-0.40, 0.27] |
|  |  |  |  |  |  |  |  |  |  |  |  |  |
| *Personality measures* |  |  |  |  |  |  |  |  |  |  |  |  |
| YSR-Anxious/Depressed | 125 | 10.20 | 4.80 |  | 68 | 6.81 | 4.47 | 4.78 | 191 | <.001 | <.001 | 0.72 [0.36, 1.08] |
| YSR-Withdrawn/Depressed | 125 | 5.28 | 3.21 |  | 68 | 4.29 | 2.83 | 2.12 | 191 | .035 | .136 | 0.32 [-0.03, 0.67] |
| YSR-SomaticComplaints | 125 | 5.98 | 3.34 |  | 68 | 3.63 | 2.77 | 4.92 | 191 | <.001 | <.001 | 0.75 [0.38, 1.11] |
| YSR-SocialProblems | 125 | 4.94 | 3.02 |  | 68 | 4.06 | 2.98 | 1.93 | 191 | .055 | .145 | 0.29 [-0.06, 0.64] |
| YSR-ThoughtProblems | 125 | 5.06 | 3.58 |  | 68 | 3.94 | 3.45 | 2.09 | 191 | .038 | .136 | 0.32 [-0.04, 0.67] |
| YSR-AttentionProblems | 125 | 6.94 | 3.11 |  | 68 | 6.71 | 3.11 | 0.49 | 191 | .626 | .683 | 0.07 [-0.28, 0.42] |
| YSR-Rule-BreakingBehavior | 125 | 3.97 | 3.32 |  | 68 | 5.06 | 4.57 | -1.89 | 191 | .060 | .145 | -0.29 [-0.64, 0.07] |
| YSR-AggressiveBehavior | 125 | 8.86 | 4.51 |  | 68 | 8.06 | 4.65 | 1.16 | 191 | .248 | .350 | 0.18 [-0.18, 0.53] |
| DERS-Non acceptance | 121 | 2.22 | 0.81 |  | 66 | 2.02 | 0.76 | 1.64 | 185 | .103 | .205 | 0.25 [-0.11, 0.61] |
| DERS-Goals | 121 | 3.08 | 0.90 |  | 66 | 2.93 | 0.91 | 1.08 | 185 | .282 | .376 | 0.17 [-0.19, 0.52] |
| DERS-Impulse | 121 | 2.34 | 0.83 |  | 66 | 2.09 | 0.76 | 2.02 | 185 | .045 | .136 | 0.31 [-0.05, 0.67] |
| DERS-Awareness | 121 | 2.71 | 0.75 |  | 66 | 2.64 | 0.61 | 0.65 | 185 | .519 | .593 | 0.10 [-0.26, 0.46] |
| DERS-Strategies | 121 | 2.40 | 0.89 |  | 66 | 2.11 | 0.86 | 2.14 | 185 | .033 | .136 | 0.33 [-0.03, 0.69] |
| DERS-Clarity | 121 | 2.57 | 0.83 |  | 66 | 1.95 | 0.52 | 5.48 | 185 | <.001 | <.001 | 0.84 [0.47, 1.21] |

*Note*: Note: n: sample size; M: mean; SD: standard deviation; t: t-statistic from the independent samples t-test; df: degrees of freedom for t; p: p-value for the t-test; p-adj: false discovery rate-adjusted p-value for the t-test; d: Cohen's d and its 95% confidence interval; RT: reaction time; AS= Antisaccade; Emo = Emotional Go-No Go; YSR = Youth Self report; DERS = Difficulties in Emotion Regulation Scale
